# Public Patterns and Determinants of Antibiotic Self-Medication and Antibiotic Knowledge in Southern Jordan

**DOI:** 10.3390/antibiotics13010098

**Published:** 2024-01-19

**Authors:** Alaa Al-Tarawneh, Tasneem Ali, Ghaith M Al-Taani

**Affiliations:** 1 Department of Allied Medical Sciences, Karak University College, Al-Balqa Applied University, Karak 19117, Jordan; ala.altarawneh@bau.edu.jo (A.A.-T.); tasneem.ali@bau.edu.jo (T.A.); 2 Department of Clinical Pharmacy and Pharmacy Practice, Faculty of Pharmacy, Yarmouk University, Irbid 21163, Jordan

**Keywords:** demographic, knowledge, self-medication, antibiotics, south Jordan

## Abstract

Antibiotic self-medication, which refers to acquisition and using antibiotics to treat infections based on personal experience and/or without a doctor’s advice or prescription, is a significant public health issue jeopardizing patient health outcomes. The purpose of the present cross-sectional online survey was to assess the frequency of self-medication among the general public in various geographical locations in southern Jordan, as well as to examine the determinants to self-medication. The survey was distributed through several social media networks over the period November–December 2022, and included demographic information as well as items related to the use and abuse of antibiotics, information sources about antibiotics, the duration of use of antibiotics, and assessment of the public knowledge about appropriate antibiotic use. Inferential analysis, such as the Chi-Square test and logistic regression, were adopted to assess the associations between the different variables with self-medication. A total of 984 respondents were enrolled in the study. Of these, 752 had been using antibiotics during the last year. However, the self-medicating cases were 413 of the 752. The main source of information about the utilization of antibiotics among participants in the survey was pharmacists. The participants commonly (36.0%) tended to use antibiotics until the symptoms disappeared. Nearly half of the respondents reported usually taking antibiotics for treating a runny nose (rhinorrhea). The logistic regression analysis indicated that self-medication with antibiotics was significantly associated with female gender (*p*-value < 0.001), low educational level (*p*-value = 0.014), rural living location (*p*-value 0.003), no health insurance (*p*-value = 0.001) and occupation (*p*-value = 0.005). Meanwhile age had no significant relationship to self-medication. Finally, the results revealed poor understanding of key appropriate antibiotic usage, which inevitably influences self-medication practice. It is crucial to come up with several programs and governmental policies to suppress widespread antibiotic self-medication as it will affect the health of future generations of Jordanian citizens.

## 1. Introduction

Self-medication with antibiotics is defined as acquiring and taking antibiotics to self-diagnose and medicate infectious diseases without consultation of a medical practitioner [1]. These antibiotics are not prescribed by physicians, but are obtained by sharing drugs with relatives, using leftover drugs stored at home, or by purchasing medicine(s) using an old prescription [2]. Self-medication with antibiotics is considered a global public health problem in developing countries [3]. For example, the rate of antibiotic self-medication among university students in Karachi, Pakistan, reached 80.4%, and it reached 68.1% among citizens living in urban areas [4,5]. In general, the prevalence of antibiotic self-medication is higher in developing countries compared to that in developed countries. The reason is that antibiotics are sold without a prescription in most developing countries, whilst they are sold in most developed countries with a prescription [6]. Nonetheless, the practice is also found in developed countries, for example Russia has a high percent of misusing antibiotics (up to 83.6%), and the figure ranges between 14% and 26% in Latin America [7,8,9]. Despite this, some antibiotics may be bought over the counter for mild illnesses as responsible use. 

Antibiotics being misused through self-medication may lead to therapeutic failures, medicine toxicity, and it could also harm individuals by increasing the risk of bacterial resistance [9]. Following the widespread usage of non-prescribed antibiotics, bacteria are becoming increasingly resistant to several types of conventional antibiotics [10]. This bacterial resistance exemplifies one of the most threatening and challenging health problems in recent times, [11,12] and developing countries are greatly affected by multidrug-resistant bacteria. For example, in 2013, the number of infections with multidrug-resistant tuberculosis skyrocketed in those countries to approximately 480,000 cases [13]. On the other hand, in developed regions like America and Europe, methicillin-resistant *Staphylococcus aureus* (MRSA) has caused the death of around 50,000 people [14]. Locally, much research has noted that *Streptococcus pneumoniae* is becoming more resistant to medications, making treatment more difficult [15,16]. Further, given the high mortality and morbidity rates associated with infectious diseases, this dramatically impacts the community’s economic stability [17]. 

In order to mitigate and avoid the consequences of this phenomenon, there must be other measures, along with government laws and financial penalties. For instance, pharmacies sell antibiotics without a doctor’s prescription, despite the fact that it is illegal to dispense antibiotics without one [18]. So, it is crucial to stress that the applicability of these laws must be a priority for policymakers. In order to prevent the community from dispensing antibiotics without a prescription, lawmakers must try to understand the factors that push citizens to practice self-medication; working to solve them is not considered a tangible solution. It is also important that work has to be done to create a deterrent that prevents citizens from this practice, as well as increasing the cultural awareness of the danger in dispensing antibiotics without a prescription and its effects on the present and future. Social media could help to play a role, as there are a huge number of users who could provide educational clips, hold educational lectures in an interesting way, and provide credible advice to patients about dispensing antibiotics. However, these behaviors may be suppressed by the efforts of policymakers, citizens and drug dispensers [19].

Several factors regarding self-medication with antibiotics have been recognized, which are typically related to low levels of education, an easy access to antibiotics, prior experience with comparable symptoms, and poverty [20]. The widespread problem of inappropriate utilization of antibiotics and non-prescription drugs is approaching alarming levels in Jordan [21]. Thus, the number of health problems related to self-treatment with antibiotics has been expected to grow in the near future. A review of previous studies has evaluated the rational use of antibiotics in northern Jordan, and suggests that the act of self-medication with antibiotics, demographic factors, and a lack of knowledge form a barrier to the knowledge of safe and appropriate use of antibiotics [22,23]. However, these relationships need to be thoroughly investigated in southern Jordan. Consequently, this study aimed to underscore the prevalence of self-medication practices among the population in southern Jordan. 

## 2. Methodology

### 2.1. Study Design

The present study comprised a descriptive-analytic cross-sectional survey to investigate the usage of antibiotics in southern Jordan at the community level. Between November and December 2022 (inclusive), the survey link was posted on social media platforms like WhatsApp, Facebook, and Twitter using the Google Forms service. Additionally, friends were requested to assist in distributing it to their relatives and friends. To ensure the quality of data collection, one response per participant and “required” answers were the settings for the Google form used. The survey was conveniently distributed to adults over the age of 18. Lay people who received invitations were requested to participate in the study through the first section of the survey, which included pertinent information about the study and what was involved, the voluntary nature of the survey, and that participants might withdraw from the study at any time. Those who accepted to participate were deemed to have given their consent, and the survey questions were then revealed to them. For those who did not consent to participate, the survey had ended and they would not have access to the survey. The Yarmouk University institutional review board examined the current study, found no ethical issues with doing the research, and permission to carry out the research was granted (Reference number: IRB/2023/168). 

### 2.2. Instrument

The survey was composed of several parts. The first part included demographic information such as gender (male, female), age (18–27, 28–37, 38–47, 48–57, >57 years), region (rural, urban), having health insurance (yes, no), the highest educational qualification attained (basic education, secondary education, technical diploma, bachelor’s degree, master’s/PhD degree), employment status (professional, governmental, manual, unemployed, student, housewife).

The second part of the survey included questions regarding the use/abuse of antibiotic therapy, such as ‘Did you take any antibiotics during 2022? (yes, no) If you used antibiotics during 2022, what was your source of antibiotic? (No, I did not. Yes, it was prescribed by a physician. Yes, I got it from a pharmacy. Yes, I took antibiotics that have remained from an old drug stored at home. Yes, I got it from my friends.) In general, where do you usually obtain your information about utilizing antibiotics? (physician, pharmacist, leaflet, previous experience, friends) Usually, how long do you take antibiotics? (1–3 days, 4–7 days, >7 days, until symptoms disappear) For what symptoms do you usually take the antibiotics? (runny nose, flu, pharyngitis, skin wounds, diarrhea, acne) 

The third part of the survey measured the public knowledge about appropriate antibiotics using six items (antibiotics are effective against viral infections, antibiotics increase the speed of recovery from colds, the utilization of antibiotics should last till the symptoms disappear regardless of the time length, antibiotics can distinguish between bacterial flora that normally live in the human body and pathogenic bacteria that cause diseases, self-prescribed antibiotics do not have health consequences, and the human body can become resistant to antibiotic after a period of time).

### 2.3. Validity and Reliability

This survey was developed based on a comprehensive literature review of previous studies [18,24]. To confirm the accuracy of the translation, the survey questions taken from the literature were first translated into Arabic and then independently back translated into English. Any concerns identified were addressed. Item development was also aided by brainstorming sessions and discussion among the research team. The study was conducted in Arabic, which is Jordan’s official language.

Face and content validity were examined in the survey conducted by review by faculty members in the Faculty of Pharmacy at Yarmouk University. The faculty members are pharmacists with a postgraduate degree in pharmacy (MSc or PhD), experience in clinical practice, and knowledge of this field of study. The survey was improved using feedback from these reviewers. The current survey instrument’s content validity index was evaluated using input from four faculty members. The content validity index was 1 (indicating that the majority of faculty either judged each item as quite relevant or relevant), confirming the face and content validity of the instrument. 

To assess the validity of the instrument, the factor analysis was conducted using principle component analysis (PCA). The sample was sufficient as indicated by the Kaiser-Meyer-Olkin (KMO) value of 0.505 (more than >0.5 cut-off point). The Bartlett’s test of sphericity yielded a *p*-value of 0.0001, demonstrating the suitability of factor analysis. Through the scree plot, the factor analysis preserved four factors that are congruent with the survey instrument’s domains. Of the overall variation, 56.9% is explained by the components in total. The instrument’s domains had little association with one another. The instrument’s domain was confirmed by the high item loading. Just a few items showed low commonality.

Pilot distribution: By performing a pilot distribution on a small sample of 30 individuals, the thoroughness and clarity of the survey were assessed. The required time to complete the survey was also assessed, and did not exceed 15 min. The input from the pilot distribution revealed that the survey parts were understood and clear, and minor alterations were made. Results from the pilot distribution were not included in the full data analysis. In this study, the Cronbach’s alpha value was 0.73, which is considered as an acceptable value according to Taber [25]. Sample size calculation was carried out using Raosoft online sample size calculation, which revealed a minimum sample size of 384 participants based on 5% margin of error and 95% confidence level.

### 2.4. Data Analysis

Standard statistical methodologies were used to assess the patterns of antibiotic self-medication. The analysis was carried out using SPSS* V21 software. Descriptive statistics were used to analyze the data, including measuring the characteristics of the participants such as gender, age, and education, in frequency tables and calculating means. Inferential analysis, including the Pearson Chi-Square test (χ2) and logistic regression, were adopted to demonstrate the significant relationships between the variables with self-medication. In all analyses the significance level was set as alpha ≤0.05. 

## 3. Results

### 3.1. Characteristics of the Survey Respondents

A total of 984 individuals responded to the survey from different regions of southern Jordan. There were more female participants (60.8%) than males, and 47.3% were within the 18–27 years’ age category. The responses were slightly more from the rural region of south Jordan (65.4%) than urban areas. Among the participants, 83.5% had health insurance. Most of the participants in the current study held a bachelor’s degree (47.3%) and only 1.3% had basic education. A total of 24.8% of the included respondents were unemployed and only 7.1% of the included respondents had governmental jobs. The characteristics of the survey respondents are represented in Table 1. 

### 3.2. Prevalence of Using Antibiotics

**Figure 1:**
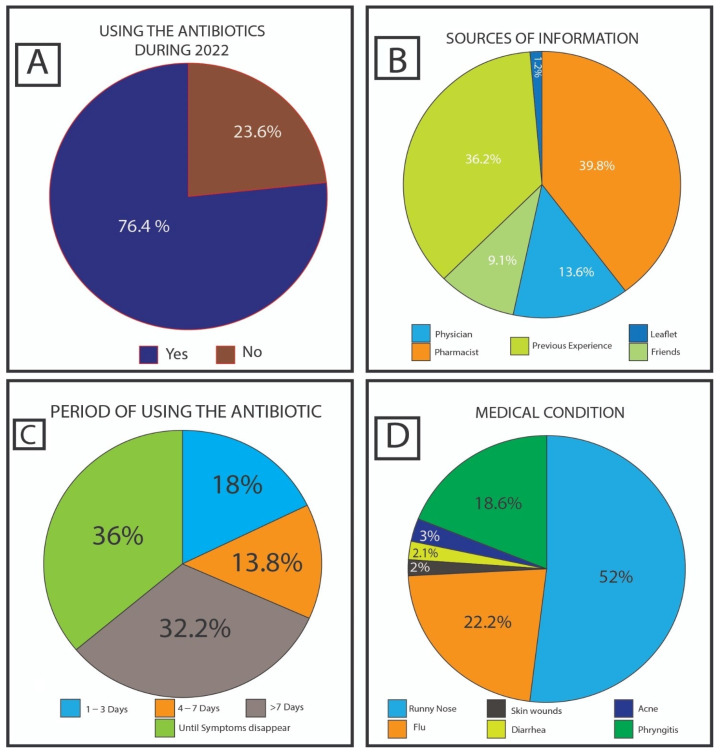
Answers to the questions: (**A**) “Did you take any antibiotics during 2022?” (**B**) “Usually, how long do you take antibiotics?” (**C**) “Where do you usually obtain your information about utilizing antibiotics?” (**D**) “For what symptoms do you usually take the antibiotics?”.

The survey included 984 participants. A total of 76.4% (752 of the 984) participants used antibiotics during the last year, as shown in Figure 1A. The analysis of questionnaire data indicated that pharmacists were commonly (39.8%) utilized as a source of information during routine service to educate the lay public. On the contrary, previous experience, physicians, and friends play a lower role in providing medical information about antibiotics, represented by 36.2%, 13.6%, and 9.1%, respectively. Leaflets had the smallest role, i.e., not exceeding 1.2%, as shown in Figure 1B. A total of 64.2% of the participants tended to use antibiotics for more than a week or until fully recovered, while 18.0% of respondents used the antibiotics for 1–3 days (the shortest period) and the rest (13.8%) usually used the drug between 4 to 7 days. Full details are shown in Figure 1C. The most common medical conditions for which the participants use antibiotics were runny nose at about 50.0%, with around 20.0% for both flu and pharyngitis. In addition, diarrhea, acne, and skin wounds were represented at a lesser extent, none exceeding 3.0% [see Figure 1D]. 

### 3.3. The Impact of Socio-Demographic Factors on Self-Medication Practice

The survey included 984 participants, but this section only takes into account the 752 participants who took antibiotics within the previous year. Of these, the self-medicating rate was 54.9% (413), while 45.1% (339) handled their cases using prescribed antibiotics (rational use). Table 2 reveals the association between self-medication with antibiotics and socio-demographic variables using Chi-square analysis, whereas Table 3 shows a logistic regression model of the socio-demographic variables as potential predictors and the outcome variable that was the self-medication represented by with odds ratios and 95% CIs. 

Females tended to misuse antibiotics more than males, using Chi-square analysis (*p*-value < 0.001), as well being nearly twice as likely to misuse these antibiotics than males (AOR = 2.207; 95.0% CI: 1.559–3.126) [see Table 3]. The relationship between age and self-medication behavior was statistically not significant (*p* = 0.067), even after adjustment for gender, education level, location, occupation, and insurance coverage using the logistic regression model (Table 2). The majority of participants (66.1%) over the age of 57 years used self-medication. Additionally, participants between the ages of 28–37 years and 48–57 years, respectively, utilized self-medication of antibiotics at a rate of about 54.0%. The percentage of self-medication of antibiotics for those between the ages of 18 and 27 years was 51.5% (Table 2). 

Education level clearly demonstrated a significant relationship withself-medication rates (*p*-value = 0.001). Starting from basic education with the highest self-use proportions at 75.0%, these rates continuously declined in participants who had completed secondary education, technical diploma, bachelor’s degree, and MSc or PhD at 64.0%, 52.5%, 51.5%, and 38.3%, respectively. 

Participants from rural areas tended to self-use antibiotics in higher proportions, about 60.0%, compared with those from urban areas who wrongly used antibiotics at approximately 46.0% (*p*-value < 0.001). The logistic regression model revealed that the possibility of self-medication in rural participants was about 1.7 times more likely compared to urban participants as shown in Table 3. The prevalence of self-medication was 68.4% in participants who were not covered by health insurance which is higher than participants who were covered by health insurance (52.5%; *p* = 0.002). Similar trends were evident with the logistic regression model that revealed that the possibility of self-medication was about 2.6 times higher for those without health insurance. 

It is generally noted that job types have a statistically significant relationship with self-medication rates, having a *p*-value that equals 0.005. The highest proportion of self-medication was reported among housewives (65.5%). Thus, the proportion of self-medication among all respondents’ groups was lower than that of housewives, as shown in Table 2. The second highest ranking was the unemployed group at 58.0%.

### 3.4. Knowledge of Antibiotics Use and Resistance

Table 4 shows six items that were used to measure respondents’ knowledge about appropriate antibiotic use and resistance. It utilizes “correct and incorrect” statements about the most common misconceptions about antibiotic usage. By answering ‘correct’ to two major misconceptions, most of the participants believed that they could take antibiotics to treat conditions caused by viral infections such as the common cold. About 70.0% of respondents use antibiotics until symptoms subside, no matter whether it is a short or long period of time. Also, 77.6% of respondents do not know that taking antibiotics for a long period of time may affect bacterial flora that normally live in their bodies because antibiotics are not able to distinguish between normal flora and pathogenic bacteria. Surprisingly, 64.6% of respondents responded to the sentence “The human body can become resistant to antibiotic after a period of use”, which related to bacterial resistance, incorrectly. 

## 4. Discussion

In our survey, prescribed antibiotics were used by 76.7% of the respondents during the last year, where 54.9% of the population took antibiotics without prescription. This is nearly similar to the results of the Ethiopian study that was conducted in Dessie City [26]. But it is interestingly less than other studies in some developing countries, such as 60.0% in Kenya, 82.2% in Nigeria, and 87.1% in Yemen [27,28,29].

Pharmacists were contacted as a main information source for the respondents who take non-prescribed antibiotics. In addition, pharmacists play an important role in forming the picture of public knowledge about antibiotic treatments. Abdel-Qader et al. pointed out that a high percentage of Jordanians who misuse antibiotics relied on pharmacists’ advice [30]. Therefore, pharmacists have to receive training programs and work constantly on maintaining their knowledge about antibiotics. Ranked second was a positive previous experience. It has been revealed that 21.0% of the participants re-use the same antibiotics due to their belief in the long-term effectiveness of these cures. Nevertheless, this may not be rational behavior, as antibiotics were utilized for treating a different type of infection or one that varies in severity. 

Prevalent medical conditions that were treated by using self-medicated antibiotics were rhinorrhea in more than half (51.0%) of respondents, followed by the common cold in around a quarter (22.0%) of respondents, and pharyngitis in 19.0% of respondents. This result is similar to other studies, in which all previous cases were caused by a viral infection and did not require antibiotics [31,32].

The usage of antibiotics by 14.0% of the participants lasted for 4–7 days, the average for most antibiotics, whereas 30.0% of the participants used them for three days or less. However, the greatest number of participants (34.0%) tended to continue utilizing the drugs until the symptoms disappeared. Indeed, scientific pieces of evidence support the idea that exposing infectious bacteria to sub-therapeutic levels of antibiotics, and using antibiotics for less than the supposed period can lead to an increased chance of bacterial resistance [33,34]. 

In the present study, the risk factors for self-medication were the gender, educational level, location, health insurance and occupation of the participant, while the age was not considered to have a significant effect on self-medication. 

Previous articles have documented a tendency by females toward self-medication, which has been confirmed in other previous studies [35,36,37]. On the other hand, other articles have found no significant differences between males and females in self-medicating behavior [38,39,40]. The variation between males and females toward self-medication might be linked to the higher susceptibility of females to some types of infection, such as contagious gynecological diseases, since they do not wish to visit a doctor when they suffer from such illnesses [41]. 

How often one practices self-medication and one’s education have had an effect on self-treating levels, in which the prevalence of self-medication was higher among people who have a low level of education, as seen in many studies [19,42,43]. This association is tied to people with higher education usually having more knowledge about misusing antibiotics and its consequences, particularly by increasing bacterial resistance.

According to the current study, housewives showed the highest possibility of self-medication behavior, up to 58.8%. That result was consistent with a finding conducted in Tabriz, Iran [24]. Self-medication is more common among housewives because they are more likely to share their experiences with acquaintances, increasing the spread of the idea that it is okay to obtain antibiotics without referring to physicians. Moreover, their accessibility to these drugs as a result of the availability at home is much higher. Unemployment ranked second as the major reason, attributable to the unaffordable cost of going to physicians. However, Abdel-Qader et al. reported that economic factors could negatively influence the public’s attitude toward self-medication practice [30].

Of those from a rural population, 62.5% of the respondents practiced self-treatment, while only 46.5% reported misusing in urban areas, which is found as a significant difference between rural and urban populations. This observation could be explained by unavailability of medical clinics in rural areas, unlike in urban areas, which could have resulted in their dependency on the pharmacies available in their areas. Additionally, relying on their previous positive experiences and listening to the opinions of their relatives and friends, exemplify how social life is experienced in rural places. A study in Lithuania has reported a similar relationship [44]. In contrast, an opposite trend has been investigated in another study, where urban people have demonstrated a high amount of self-medication practices [38]. 

In addition, this study suggests that health insurance plays a vital role in the usage of these cures. Participants with no health insurance are more prone to take antibiotics without prescription by 1.567%, compared with covered participants. This trend may be explained by economic considerations, since people who have medical insurance are able to refer to physicians and obtain the adequate antibiotics [45]. This finding agrees with the result of an Indonesian study [46]. 

Defects in public knowledge about appropriate antibiotics usage influences self-medication behavior. In the current study, 74.1% and 77.1% of participants believed that antibiotics are able to treat common colds and eradicate viruses, respectively, but in fact, viral infections do not require antibiotic treatment [47]. Indeed, the rational use of antibiotics can be achieved by using the right antibiotics in adequate doses for the proper amount of time [48]. However, 67.6% of participants use antibiotics until symptoms disappear, and this may mean exposing bacteria to sub-therapeutic levels, which makes bacteria more resistant to antibiotics [49]. Further, the lack of awareness regarding antimicrobial resistance was obviously seen on the questionnaire data. To sum up, a lack of information about the effectiveness of antibiotics and bacterial resistance exists in the southern part of Jordan.

One limitation of this study is that respondents without smartphones or internet access may have been unable to contribute to the study because the distribution of the survey was through online platforms. In spite of this, it is expected nowadays that this population is quite small and would not have a substantial impact on the study’s findings. Furthermore, the cross-sectional design made it difficult to detect changes in participants’ responses over time. Also, the questionnaire targeted different segments of the society who were at varied awareness levels regarding the concept of antibiotics. Nevertheless, the survey failed to ask about the participants’ level of knowledge regarding drugs that may be classified as antibiotics and as a result failed to modify the background information for the questions based on this piece of information, particularly for those who had poor health literacy. Additionally, the study failed to address the issue that some antibiotics may be bought over the counter for mild illnesses as responsible use.

## 5. Conclusions

This study evaluated the extent of self-medication with antibiotics in southern Jordan and found that, in spite of the national legislation prohibiting and fighting self-medication, antibiotics are obtained without a medical prescription in southern Jordan with relative ease. The results of this study also pointed out socio-demographic variables related to self-medication, such as female gender, low education level, living in rural areas, and housewife occupation. This study also revealed a lower level of knowledge among participants regarding antibiotic use. The results of this study also provide sufficient motivation for the government to develop several programs and governmental policies to reduce the dispensing of antibiotics that are prescribed in an unregulated manner. 

## Figures and Tables

**Table 1:** The socio-demographic characteristics of the participants.

Variables		All Participants	
		Frequency	Percent
Gender	Male	386	39.2
	Female	598	60.8
Age	18–27	569	57.8
	28–37	217	22.1
	38–47	96	9.8
	48–57	40	4.1
	>57	62	6.3
Education level	Basic education	13	1.3
	Secondary education	310	31.5
	Technical diploma	106	10.8
	Bachelor’s degree	465	47.3
	MSc or PhD	90	9.1
Region	Urban	340	34.6
	Rural	644	65.4
Health insurance	YES	822	83.5
	NO	162	16.5
Job	Professional	166	16.9
	Governmental	70	7.1
	Manual	170	17.3
	Unemployed	244	24.8
	Student	166	16.9
	Housewife	168	17.1

**Table 2:** The association between self-medication with antibiotics and socio-demographic variables.

Variables		Total n (%)	Self-Medication n (%)	Rational n (%)	*X* ^2^	*p*-Value
Gender	Male	343 (45.6)	172 (50.1)	171 (49.9)	5.807	0.016
	Female	409 (54.4)	241 (58.9)	168 (41.1)		
Age	18–27	412 (54.8)	212 (51.5)	200 (48.5)	8.79	0.067
	28–37	165 (21.9)	90 (54.5)	75 (45.5)		
	38–47	79 (10.5)	52 (65.8)	27 (34.2)		
	48–57	37 (4.9)	20 (54.1)	17 (45.9)		
	>57	59 (7.8)	39(66.1)	20 (33.9)		
Education	Basic Education	12 (1.6)	9 (75.0)	3 (25.0)	18.481	0.001
	Secondary Education	239 (31.8)	153 (64.0)	86 (36.0)		
	Technical Diploma	80 (10.6)	42 (52.5)	38 (47.5)		
	Bachelor’s Degree	361 (48.0)	186 (51.5)	175 (48.5)		
	MSc Or PhD	60 (8.0)	23 (38.3)	37 (61.7)		
Location	Urban	254 (33.8)	117 (46.1)	137 (53.9)	12.154	<0.001
	Rural	498 (66.2)	296 (59.4)	202 (40.6)		
Health insurance	Yes	638 (84.8)	335 (52.5)	303 (47.5)	9.893	0.002
	No	114 (15.2)	78 (68.4)	36 (31.6)		
Occupation	Professional	129 (17.2)	59 (45.7)	70 (54.3)	16.939	0.005
	Governmental	36 (4.8)	13 (36.1)	23 (63.9)		
	Manual	115 (15.3)	64 (55.7)	51 (44.3)		
	Unemployed	188 (25.0)	109 (58.0)	79 (42.0)		
	Student	142 (18.9)	75 (52.8)	67 (47.2)		
	Housewife	142 (18.9)	93 (65.5)	49 (34.5)		

**Table 3:** Factors associated with self-medication by antibiotics using logistic regressions.

Variable		Wald	df	*p*-Value	Adj.OR	95.0% CI (AOR)	
						Lower	Upper
Gender	Male				1 ^c^		
	Female	19.904	1	<0.001	2.207	1.559	3.126 ^a^
Age	18–27	6.547	4	0.162	1 ^c^		
	28–37	0.119	1	0.730	1.132 ^b^	0.559	2.293
	38–47	0.135	1	0.714	0.870 ^b^	0.412	1.835
	48–57	1.823	1	0.177	0.558 ^b^	0.239	1.302
	>57	0.093	1	0.761	1.158 ^b^	0.451	2.973
Education	Basic Education	12.484	4	0.014	1 ^c^		
	Secondary Education	5.214	1	0.022	0.496 ^b^	0.272	0.906
	Technical Diploma	1.481	1	0.224	0.637 ^b^	0.308	1.317
	Bachelor’s Degree	9.494	1	0.002	0.363 ^b^	0.190	0.691
	MSc Or PhD	4.143	1	0.042	0.203 ^b^	0.044	0.943
Location	Urban				1 ^c^		
	Rural	8.569	1	0.003	1.672	1.185	2.359 ^a^
Health insurance	Yes				1 ^c^		
	No	12.645	1	<0.001	2.603	1.536	4.411 ^a^
Occupation	Professional	12.413	5	0.030	1 ^c^		
	Governmental	0.512	1	0.474	0.832 ^b^	0.503	1.376
	Manual	5.136	1	0.023	0.556 ^b^	0.335	0.924
	Unemployed	2.479	1	0.115	1.878 ^b^	0.857	4.114
	Student	1.620	1	0.203	0.707 ^b^	0.415	1.206
	Housewife	3.353	1	0.067	2.154 ^b^	0.948	4.895

Adj. OR; Adjusted odds ratio; ^a^ Likelihood Ratio (LR) test; ^b^ Wald test; ^c^ reference group.

**Table 4:** Knowledge about appropriate antibiotic use and resistance.

Statements (Correct Response)	Correct Answer		Incorrect Answer	
	Frequency	Percent (%)	Frequency	Percent (%)
Antibiotics are effective against viral infections. (NO)	281	28.6	703	71.4
Antibiotics increase the speed of recovery from colds. (NO)	316	32.1	668	67.9
The utilization of antibiotics should last till disappear the symptoms regardless the time length. (NO)	294	29.9	690	70.1
Antibiotics can distinguish between bacterial flora that normally live in the human body and pathogenic bacteria that cause diseases. (NO)	220	22.4	764	77.6
Unprescribed antibiotics does not have health consequences. (NO)	479	48.7	505	51.3
The human body can become resistant to antibiotic after a period of use. (YES)	348	35.4	636	64.6

## Data Availability

Further inquiries about the data sets may be directed to the corresponding author.
